# Flecainide induces a sustained countercurrent dependent effect on RyR2 in permeabilized WT ventricular myocytes but not in intact cells

**DOI:** 10.3389/fphar.2023.1155601

**Published:** 2023-04-12

**Authors:** Emma J. Steer, Zhaokang Yang, Moza M. Al-Owais, Hannah M. Kirton, Edward White, Derek S. Steele

**Affiliations:** Faculty of Biological Sciences, School of Biomedical Sciences, University of Leeds, Leeds, United Kingdom

**Keywords:** heart, myocyte, calcium, waves, confocal, arrhythmias, flecainide, ryanodine receptor

## Abstract

**Background and purpose:** While flecainide is now an accepted treatment for arrhythmias associated with catecholaminergic polymorphic ventricular tachycardia (CPVT), its mechanism of action remains controversial. In studies on myocytes from CPVT mice, inhibition of proarrhythmic Ca^2+^ waves was initially attributed to a novel action on the type-2 ryanodine receptor (RyR2). However, subsequent work on wild type (WT) myocytes questioned the conclusion that flecainide has a direct action on RyR2. In the present study, the effects of flecainide were compared in intact and permeabilized WT myocytes.

**Experimental approach:** Intracellular Ca^2+^ was measured using confocal microscopy in intact or saponin permeabilized adult rat ventricular myocytes (ARVM). In some experiments on permeabilized cells, flecainide was studied following partial inhibition of the sarcoplasmic reticulum (SR) counter-current.

**Key results:** Flecainide induced sustained changes Ca^2+^ sparks and waves in permeabilized ARVM, which were comparable to those reported in intact or permeabilized myocytes from CPVT mice. However, a relatively high level of flecainide (25 μM) was required to induce these effects. Inhibition of the SR counter-current potentiated the effects of flecainide on SR Ca^2+^ waves. In intact field stimulated ARVM, prolonged exposure to 15 μM flecainide decreased wave frequency but RyR2 dependent effects on Ca^2+^ sparks were absent; higher drug concentrations blocked field stimulation, consistent with inhibition of Nav1.5.

**Conclusions and implications:** In intact ARVM, the absence of effects on Ca^2+^ sparks suggests that the intracellular flecainide concentration was insufficient to influence RyR2. Wave inhibition in intact ARVM may reflect secondary effects of Nav1.5 inhibition. Potentiation of flecainide’s action by counter-current inhibition can be explained if transient polarization of the SR membrane during SR Ca^2+^ release facilitates its action on RyR2.

## 1 Introduction

In a landmark study, Knollmann and colleagues were the first to demonstrate that the class 1C antiarrhythmic agent flecainide can prevent ‘catecholaminergic polymorphic ventricular tachycardia’ (CPVT) arrhythmias in a mouse model of the disease and in a small patient cohort (Watanabe et al., 2009). In subsequent clinical trials, flecainide was effective at suppressing arrhythmias in CPVT patients, including those resistant to conventional treatment with a high dose β-blocker (Khoury et al., 2013; van der et al., 2011; Wanguemert et al., 2018; Kannankeril et al., 2017).

While the clinical efficacy of flecainide in CPVT patients is now accepted, its antiarrhythmic mechanism remains controversial (Sikkel et al., 2013a; Sikkel et al., 2013b; Steele et al., 2013). Flecainide is a well characterized use-dependent inhibitor of the sodium channel (NaV1.5) (Salvage et al., 2018). However, its primary action in CPVT was initially attributed to direct modulation of RyR2 and consequent suppression of spontaneous SR Ca^2+^ waves (Hilliard et al., 2010; Savio-Galimberti and Knollmann, 2015). Such an effect would prevent delayed afterdepolarizations (DADs), which occur when the electrogenic Na^+^/Ca^2+^ exchanger (NCX) extrudes Ca^2+^ released during spontaneous SR Ca^2+^ release. Should a spontaneous wave occur despite intracellular suppression by flecainide, NaV1.5 inhibition would reduce the probability of an action potential being triggered, providing a secondary level of protection (Liu et al., 2011).

Evidence supporting an effect of flecainide on RyR2 was first obtained in paced myocytes from CPVT mice, where it decreased the incidence of spontaneous SR Ca^2+^ waves during β_1_-adrenergic stimulation (Watanabe et al., 2009). Importantly, flecainide also inhibited Ca^2+^ waves in *permeabilized* myocytes from CPVT mice or WT adult rat ventricular myocytes (ARVM), in which sarcolemmal ionic fluxes are entirely absent (Hilliard et al., 2010; Galimberti and Knollmann, 2011). A decrease in the amplitude and width of Ca^2+^ sparks was proposed as the primary event leading to inhibition of Ca^2+^ waves, which involve salutatory propagation of localized Ca^2+^ release between junctional RyR2 clusters (Hilliard et al., 2010). Open-state block of isolated channels incorporated into lipid bilayers was cited as further evidence of a direct effect of flecainide on RyR2 (Hilliard et al., 2010).

A number of subsequent studies questioned whether flecainide has a direct effect on RyR2, e.g., Sikkel *et al* investigated the effects of flecainide on ARVM in which waves were induced by abrupt cessation of rapid pacing (Sikkel et al., 2013a). Although flecainide decreased Ca^2+^ wave frequency, the previously described effects on spark properties were absent and the authors attributed its action to inhibition of NaV1.5. In a subsequent study, flecainide had no apparent effect on isolated RyR2 channels incorporated into lipid bilayers under conditions considered physiologically relevant (Bannister et al., 2015). The same study found no effect of flecainide on SR Ca^2+^ regulation in permeabilized ARVM. Further work on ARVM addressed the effects of flecainide derivatives introduced *via* patch pipettes, but again the characteristic changes in SR Ca^2+^ regulation reported in CPVT myocytes were not observed (Bannister et al., 2016).

These studies challenging the concept that flecainide modulates RyR2 have several potential weaknesses. First, it is questionable whether definitive conclusions can be drawn from lipid bilayer experiments alone, because the conditions differ significantly from the physiological state, including in some cases the presence of the RyR2 agonist EMD4100 (Bannister et al., 2015; Bannister et al., 2016). Second, it has typically been assumed that WT ARVM should respond similarly to cells from CPVT hearts (Sikkel et al., 2013a; Bannister et al., 2015; Bannister et al., 2016). While flecainide-induced effects on RyR2 have been reported in permeabilized ARVM (Hilliard et al., 2010), the level of drug required was higher than the peak plasma concentration in patients (Mano et al., 2015), or that needed to modulate RyR2 in cells from CPVT mice (Galimberti and Knollmann, 2011; Gomez-Hurtado et al., 2015). As with other cationic drugs, flecainide accumulates within the heart (Conard and Ober, 1984; Latini et al., 1987), but it is not known whether it concentrates sufficiently within the cytosol of ARVM to influence WT RyR2.

The present study addresses RyR2-mediated effects in intact WT ARVM in an attempt to understand why previous studies have produced such discrepant findings. In addition, experiments were carried out in permeabilized ARVM to further characterize the effects of flecainide on SR Ca^2+^ waves and to investigate a recent proposal that the action of the drug may be dependent upon counter-current inhibition and transient polarization of the SR membrane during spontaneous Ca^2+^release (Kryshtal et al., 2021; Bannister et al., 2022).

## 2 Materials and methods

### 2.1 Preparation of cells and solutions

Wistar rats (150–200 g) were sacrificed in accordance with the UK Home Office Guidance on the Operation of Animals (Scientific Procedures) Act of 1986 and with local Ethics Committee approval. ARVM were isolated by collagenase and protease digestion as previously described (Yang and Steele, 2000). Intact ARVM were perfused with Tyrode’s solution containing (mM) 136.9 NaCl; 5.4 KCl; 0.5 MgCl_2_; 0.5 NaH2PO_4_; 1 HEPES; 11.1 glucose; 1.8 CaCl_2_, 20–22 C, pH 7.1. For confocal Ca^2+^ imaging of intact ARVM, cells were loaded with fluo-4 AM (6 µM) for 15-min at room temperature (20–22 C). A further 30-min was then allowed for de-esterification.

In some experiments ARVM were permeabilized by brief (20-min) exposure to saponin (10 mg/mL) in a mock intracellular solution containing (mM) 100 KCl; 5.72 MgCl_2_; 25 HEPES; 5 Na_2_ATP; 10 Na_2_CrP; 0.1 EGTA, 20–22 C, pH 7.0. Fluo-3 (15 μM, potassium salt) was included to allow changes in [Ca^2+^] to be detected using confocal microscopy. The free [Ca^2+^] was adjusted using CaCl_2_ and calculated using REACT software (Duncan et al., 1999). In all permeabilized cell experiments, the use of glassware was avoided to prevent Ca^2+^ contamination of the weakly Ca^2+^ buffered solutions. In pilot experiments where glassware was used, high and variable levels of Ca^2+^ contamination made it difficult to identify consistent effects of flecainide. When studying spontaneous SR Ca^2+^ waves, the free [Ca^2+^] of the bathing solution was ∼280 nM and [EGTA] was 0.1 mM. For experiments involving Ca^2+^ spark detection, the [EGTA] was increased to 0.35 mM to prevent formation of propagated Ca^2+^ waves (Yang and Steele, 2001). Prevention of wave formation ensures that the SR Ca^2+^ content reaches a steady state, allowing the effects of flecainide on sparks to be detected against a relatively constant background [Ca^2+^], which is optimal for automated spark detection and analysis using SparkMaster (see below). In experiments involving partial inhibition of the SR charge compensating countercurrent in permeabilized cells, KCl was replaced with cesium methanesulfonate (CH_3_O_3_SCs)**,** MgCl_2_ and with MgSO_4_. pH was adjusted with cesium hydroxide hydrate.

Unless otherwise stated, all chemicals were obtained from Sigma Aldrich. Fluo-4 AM was obtained from Life Technologies, United States, and fluo-3 from Biotium, United States.

### 2.2 Experimental setup

The experimental chamber was placed on the stage of a Nikon Eclipse TE2000-U inverted microscope with a BioRad Cellmap confocal unit. Cells were viewed *via* a ×40 oil immersion lens. The dye was excited with a 20 mW 488 nm diode laser which was attenuated to ∼7%. Line-scan confocal microscopy was used to investigate rapid changes in intracellular [Ca^2+^] in intact and permeabilized ARVM.

To investigate the effects of flecainide on spontaneous SR Ca^2+^ release in intact ARVM, quiescent cells were initially exposed to Tyrode’s solution containing 100 nM isoproterenol and either 15 µM flecainide or vehicle (ethanol, <0.01%) for a minimum of 45-min. This prolonged equilibration period reflects the fact that flecainide enters cells only slowly because it is mostly in its positively charged form at physiological pH; Previous work has shown that exposure for at least 30-min was required to observe RyR2 effects in cells from CPVT mice (Sikkel et al., 2013b). Levels of flecainide >15 µM were not used because field stimulation induced responses were progressively blocked, presumably due to inhibition of NaV1.5. As in studies on CPVT myocytes, isoproterenol was included to mimic β-adrenergic stimulation and increase the incidence of pro-arrhythmic SR Ca^2+^ waves (Hilliard et al., 2010). Following flecainide preincubation, intact ARVM were paced (field stimulation *via* platinum electrodes) for a further 10-min with a 10 ms square wave (∼50%> threshold). Stimulation was then stopped, and cells imaged in line scan mode for 80 s.

For studying Ca^2+^ waves in permeabilized ARVM, cells were initially imaged in line scan mode for 30 s during perfusion with a control solution containing fluo-3 (10 µM). The perfusion solution was then switched to a similar solution containing either flecainide (25 µM) or vehicle (ethanol, <0.01%) and line scanned for 30 s at 1-min intervals for a total of 5-min. This allowed the effect of flecainide to be studied over a more prolonged period while minimizing any effects due to laser exposure. In pilot experiments where intermittent scanning was not employed, laser exposure increased the variability of the control response, making drug effects undetectable (*not shown*).

### 2.3 Analysis of confocal images

All confocal data were viewed and analyzed using ImageJ. The automated detection of Ca^2+^sparks and measurement of their temporal and spatial properties was carried out using the SparkMaster plugin for ImageJ (Picht et al., 2007). This included spark amplitude (F/F_0_), full duration at half maximum amplitude (FDHM), full width at half maximum amplitude (FWHM) and frequency (sparks/100 μm/s). The detection threshold was set at 3.8 times the SD of the background noise above the mean background signal. Line-scan (x-t) images are presented with time (t) on the horizontal axis and distance (x) on the vertical axis. In some cases, amplitude and time course of selected spark events were indicated using line profiles. Line profiles were produced in ImageJ by positioning a narrow box (2 pixels high), centered on the peak of an individual spark. The pixel values at each time point were then averaged to produce a continuous line.

### 2.4 Statistics

Statistical analyses were performed using paired and unpaired Student’s t-tests or ANOVA with Holm-Sidak *post hoc* test or as indicated. P < 0.05 was considered statistically significant. All data were displayed as mean ± SEM. The number of cells followed by the number of animals/hearts in parenthesis is shown in figure legends.

## 3 Results

### 3.1 Effects of flecainide on Ca^2+^ waves in permeabilized ARVM

**FIGURE 1:**
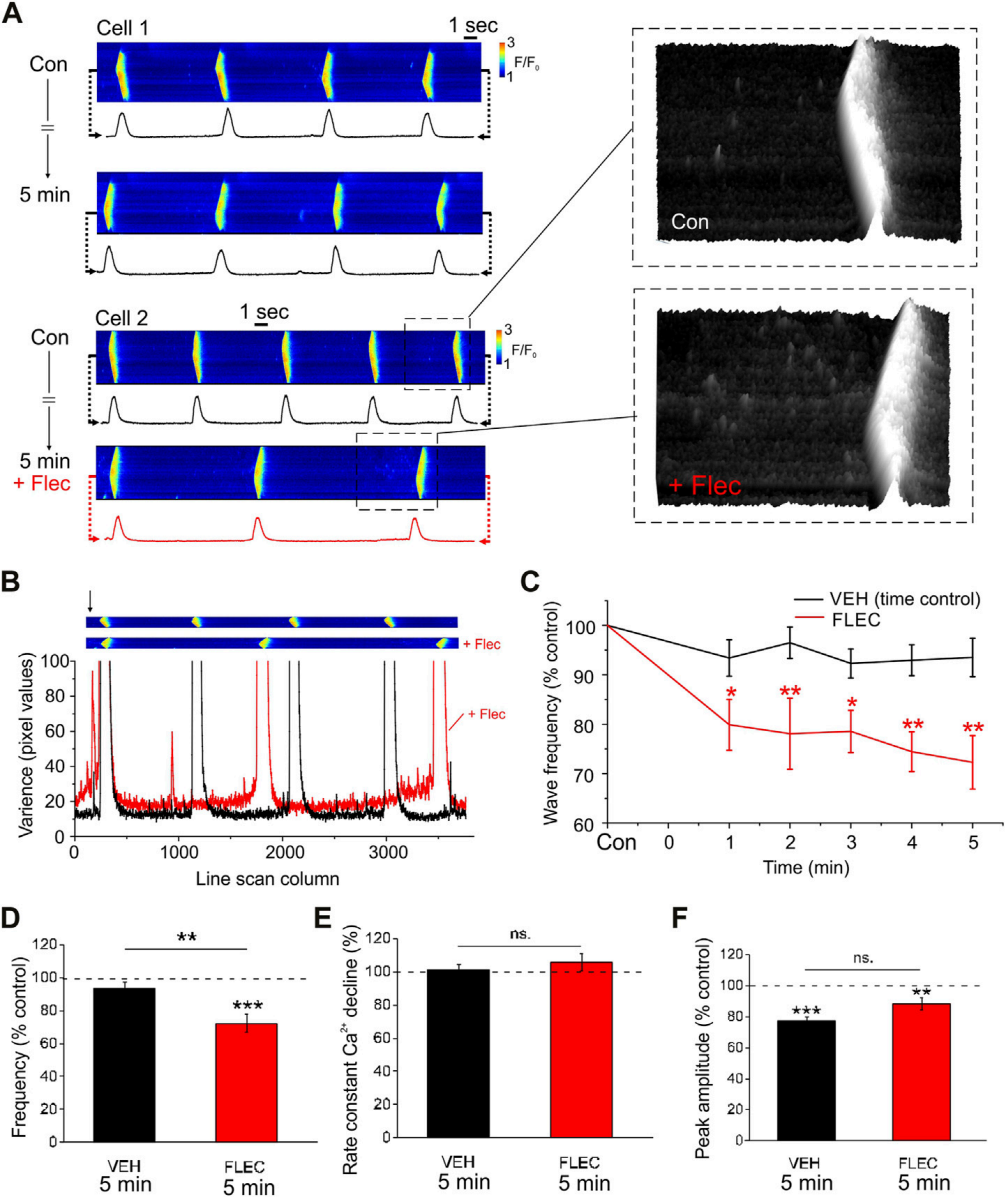
Flecainide decreases Ca^2+^ wave frequency in permeabilized ARVM **(A)**, Representative line scans of cells and corresponding line profiles under control conditions (Con) and after 5 min incubation with either vehicle (*upper*) or 25 µM flecainide (*lower*). Enlarged high contrast regions show changes in spark frequency during the period just before each wave. **(B)**, Line scan images before and after addition of flecainide (*upper*) and corresponding plot showing the variance of each column (*arrow*) of pixel values before (black) and after (red) exposure to flecainide. **(C)**, Percentage change in wave frequency as a function of time. **(D–F)**, Percentage change expressed relative to time zero control (dotted line)*,* wave frequency, the time constant for the decrease in [Ca^2+^] and wave amplitude. Repeated measures ANOVA with Holm-Sidak *post hoc* test or paired or unpaired Student’s t-tests as appropriate. * = *p* < 0.05; ** = *p* < 0.01; *** = *p* < 0.001. CON *n* = 10 (4), FLEC *n* = 12 (4).

To investigate the intracellular effects of flecainide independently from its action on sarcolemmal ion channels or resultant changes in the cytosolic environment, SR Ca^2+^ regulation was studied in permeabilized ARVM. Each cell was initially imaged during perfusion with a weakly Ca^2+^ buffered ‘intracellular’ solution containing fluo-3. Either flecainide or the vehicle was then introduced for a further 5-min. Under control conditions there was a tendency for wave frequency to decrease slightly during the 5-min protocol (Figure 1A, upper left). However, flecainide induced a more pronounced decrease in wave frequency over the same period (Figure 1A, lower left). In the presence of flecainide, line scan images typically exhibited a higher level of noise, particularly in the period immediately before each wave (Figure 1A, right). Figure 1B shows the variance of each column of pixel values as a function of time for line scan images obtained in the same cell before and after addition of flecainide. The variance of the pixel values increased markedly just before each wave and this effect was more pronounced in the presence of flecainide. This is due in part to an increase in Ca^2+^ spark frequency but may also reflect an increase in smaller unresolvable SR Ca^2+^ release events (Zima et al., 2010).

As shown in the cumulative data, flecainide decreased wave frequency significantly after 1-min and this effect increased progressively (Figure 1C). The decrease in wave frequency at 5-min (∼25%) is comparable with that reported in permeabilized myocytes from CPVT mice under similar conditions (e.g., Supplementary data in Galimberti and Knollmann, 2011). Importantly, however, it was necessary to apply a much higher concentration of flecainide in WT ARVM than in CPVT myocytes (25 vs. 6 µM). This is consistent with previous observations that RyR2 mutations confer a higher sensitivity to the drug, although species differences likely also play a role (Savio-Galimberti and Knollmann, 2015).

The decrease in frequency at 5-min (Figure 1D) occurred without a change in the rate of decline of the Ca^2+^ transients (Figure 1E), which is consistent with previous studies suggesting that flecainide does not affect SR Ca^2+^ uptake *via* SERCA. In both flecainide and vehicle treated cells, wave amplitude decreased slightly relative to the time zero control value (Figure 1F). However, there was no significant difference between the amplitude of Ca^2+^ waves in flecainide or control cells after 5-min. Hence, in permeabilized ARVM, flecainide appears to delay the formation of spontaneous Ca^2+^ waves, without having other major effects on SR Ca^2+^ handling.

### 3.2 Effects of flecainide on Ca^2+^ sparks in permeabilized ARVM

**FIGURE 2:**
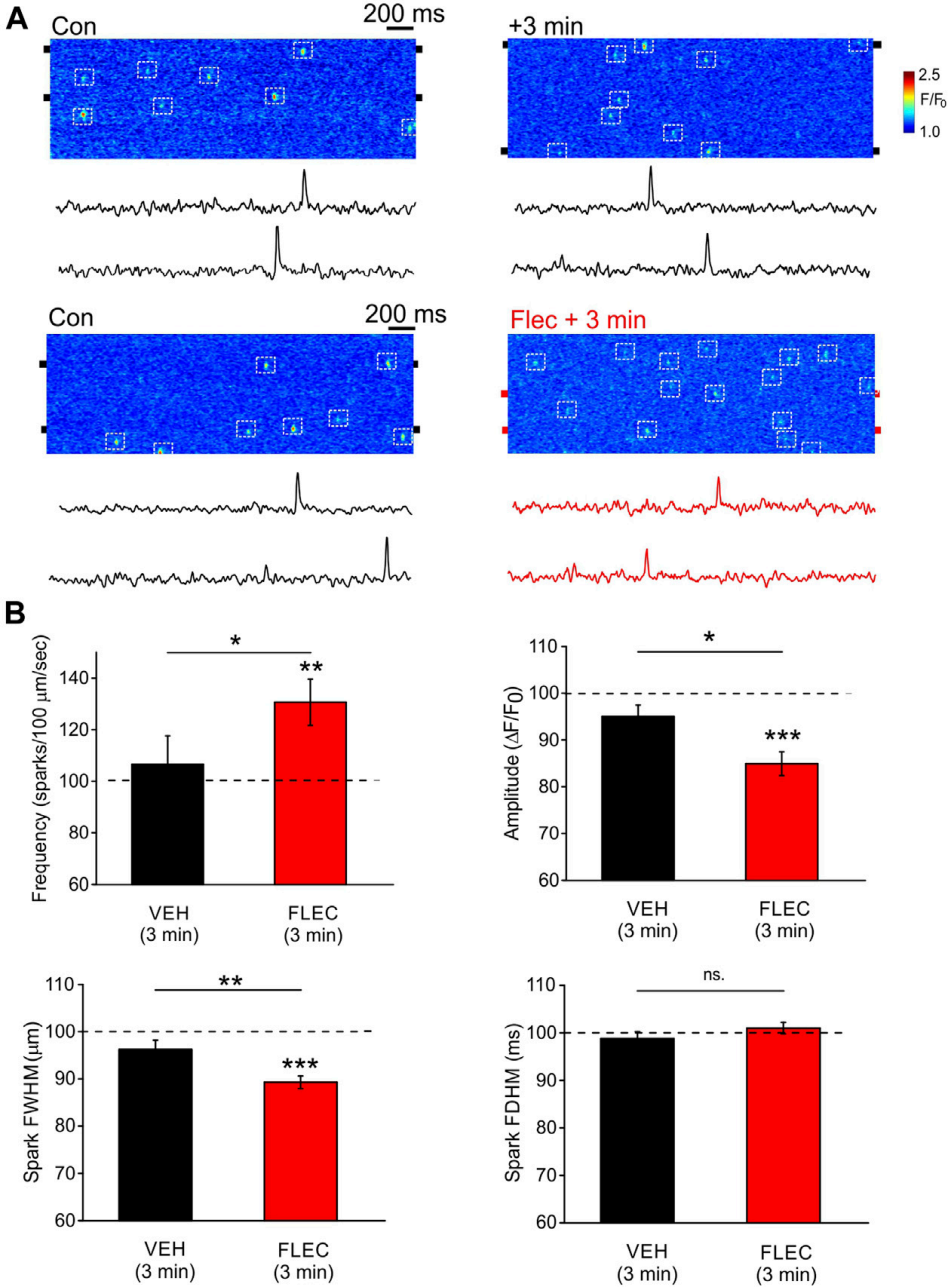
Effects of flecainide on Ca^2+^ spark properties in permeabilized ARVM **(A)**, Representative line scan images and accompanying line profiles from two ARVM under control conditions and then in the corresponding cell 3 after-minutes incubation with either vehicle (*upper*) or 25 µM flecainide (*lower*). The position of the box (2 pixels high) used to produce the line profile was centered on the pairs of black ‘tabs’ shown on either side of each image. (**B)** *,* Cumulative data showing the percentage change in spark frequency, amplitude, width and duration expressed relative to the time zero control (dotted line). Paired and unpaired Student’s t-tests as appropriate. * = *p* < 0.05; ** = *p* < 0.01; *** = *p* < 0.001. CON *n* = 11 (4), FLEC *n* = 31 (5).

To study the effects of flecainide on Ca^2+^ sparks under more controlled conditions, permeabilized ARVM were exposed to intracellular solutions with a higher Ca^2+^ buffer capacity (0.36 mM EGTA), which prevents localized Ca^2+^ sparks propagating to form waves (Yang and Steele, 2000). Line scan images and associated line profiles from two ARVM are shown at time zero and then 3-min after introduction of either the vehicle or flecainide (Figure 2A). Under control conditions in the presence of the vehicle alone, there was no apparent change of spark frequency after 3-min. However, flecainide increased spark frequency over the same period. The cumulative data show that the vehicle alone had no significant effect on any of the measured spark parameters (Figure 2B). In contrast, flecainide significantly increased spark frequency, while decreasing both spark amplitude and width. There was no effect of flecainide on spark duration. These data show that despite the high level of flecainide required, the effects on sparks in ARVM are qualitatively similar to findings in intact CPVT myocytes (Hilliard et al., 2010).

### 3.3 Counter-current inhibition potentiates the effects of flecainide in permeabilized ARVM

Work on isolated RyR2 incorporated into lipid bilayers has shown that flecainide predominantly exerts a blocking action on RyR2 when the ionic flux is cytosolic to luminal (Bannister et al., 2015). This was interpreted as evidence that flecainide has no RyR2 blocking action under physiological conditions, when Ca^2+^ flows from the SR lumen to the cytosol. However, such experiments typically involve the application of a potential difference across the lipid bilayer. Hence, an alternative interpretation is that flecainide-induced RyR2 block requires the SR lumen to be negative with respect to the cytosol. This condition is met when SR Ca^2+^ release transiently polarizes the SR, leading to charge compensating counter-current fluxes (e.g., K^+^, Cl^−^, Mg^2+^). Conversely, during Ca^2+^ uptake, the SR lumen would be expected to become transiently positive with respect to the cytosol.

**FIGURE 3:**
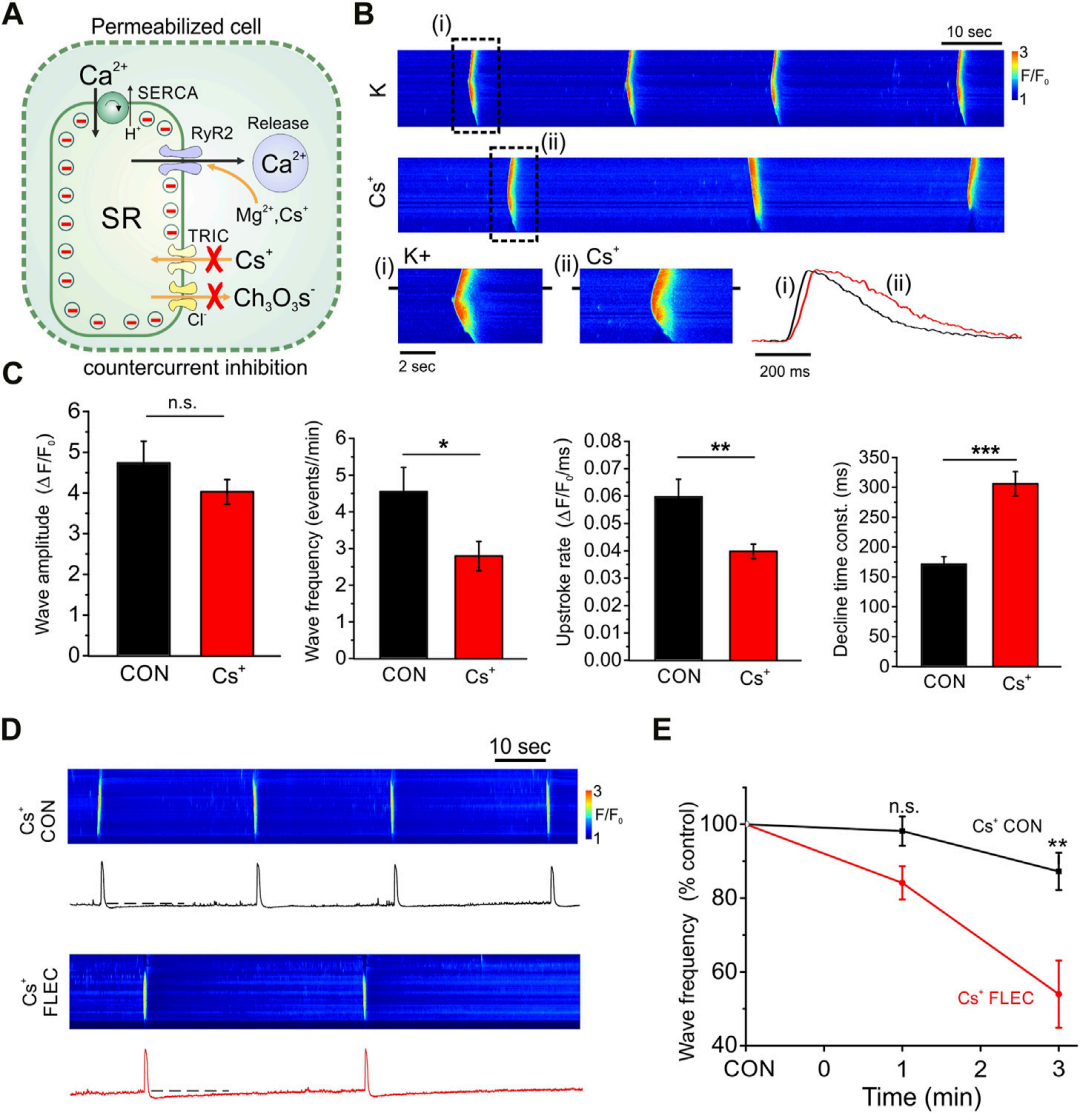
Effects of countercurrent inhibition on SR Ca^2+^ regulation and the action of flecainide. **(A)**, Diagram shows permeabilized cell in which counter-currents during SR Ca^2+^ release are inhibited by substitution of K^+^ and Cl^−^ with Cs^+^ and Ch_3_O_3_S^−^ ions, which have a low permeability through SR K^+^ (TRIC) channels and Cl^−^ channels respectively. Counter-current movement (including Cs^+^) can still occur *via* RyR2 and SERCA exhibits counter transport of H^+^. Partial counter-current inhibition would be expected to increase transient SR polarization during Ca^2+^ influx or efflux, thereby opposing Ca^2+^ movements across the SR membrane. **(B)**, Line scan images and corresponding line profiles from the same cell before (i) and after (ii) counter-current inhibition, showing a decrease in Ca^2+^ wave frequency and slowing of both Ca^2+^ release and reuptake. **(C)**, Cumulative data showing effects of counter-current inhibition on Ca^2+^ wave amplitude, frequency, upstroke rate and the time constant of the descending phase. **(D)**, Line scan images and corresponding line profiles from the same cell before and after introduction of flecainide during constant counter-current inhibition. **(E)**, Cumulative data showing time dependent effects of flecainide on Ca^2+^ wave frequency during counter-current inhibition. Cs^+^ control (Cs^+^ CON) n = 13 (5), Cs^+^ flecainide (Cs^+^ FLEC) n = 13 (5), **p < 0.001.

To test the possible involvement of transient SR polarization, permeabilized cells were exposed to solutions in which K^+^ was substituted with Cs^+^ and Cl^−^ for Ch_3_O_3_S^−^(Figure 3A); SR chloride channels are relatively impermeant to Ch_3_O_3_S^−^ (Rousseau et al., 1988). Similarly SR K^+^ (TRIC) channels are relatively impermeant to Cs^+^ ions (Guo et al., 2013). Under these conditions some counter-current will remain, e.g., due to movement of Mg^2+^ and Cs^+^ *via* RyR2 and H^+^, which is transported *via* SERCA. However, the reduced counter-current would be expected to increase the magnitude of any transient SR polarization with consequent effects on Ca^2+^ fluxes.

Original line scan images are shown before and after inhibition of the counter-current by ionic substitution with Cs^+^ and Ch_3_O_3_S^−^ ions (Figure 3B). Counter-current inhibition resulted in a decrease in the frequency of spontaneous SR Ca^2+^ waves and prolongation of both rising and descending phases of the Ca^2+^ transient. As shown in the cumulative data, counter-current inhibition had no major effect on wave amplitude but significantly decreased both the SR Ca^2+^ release and uptake rates (Figure 3C). As considered previously (Kawai et al., 1998), such effects are consistent with a greater transient polarization of the SR during Ca^2+^ uptake and release, which opposes the movement of Ca^2+^ in both directions.

As shown in the line scan images (Figure 3D) and the cumulative data (Figure 3E), during constant counter-current inhibition, the flecainide-induced decrease in frequency of SR Ca^2+^ waves was more than double that obtained at the same time point under control conditions (compare with Figure 1C at 3-min). Indeed, in ∼30% of cells, flecainide abolished spontaneous SR Ca^2+^ release completely within 3-min (not shown).

### 3.4 In intact ARVM flecainide decreases wave frequency but has no effect on Ca^2+^ sparks

Experiments were carried out to establish the effects of flecainide on spontaneous Ca^2+^ waves in intact field stimulated ARVM. As flecainide has a pKa of 9.3 and is 99% positively charged at physiological pH, previous work has shown that at least 30-min is needed to allow slow permeation and intracellular effects on RyR2 to be detected (Steele et al., 2013). Therefore, flecainide treated cells were initially exposed to the drug for at least 45-min before beginning the experiment and a high perfusate concentration (15 µM) was used to increase the possibility of observing RyR2 mediated effects; concentrations >15 µM progressively blocked field stimulation due to inhibition of Nav1.5 (not shown). Control (vehicle) or drug treated cells were superfused with isoproterenol (100 nM) and field stimulated at 1.5 Hz for 10-min. To minimize laser exposure, line scan imaging was initiated just before cessation of stimulation, which was typically followed by repeated spontaneous SR Ca^2+^ waves.

**FIGURE 4:**
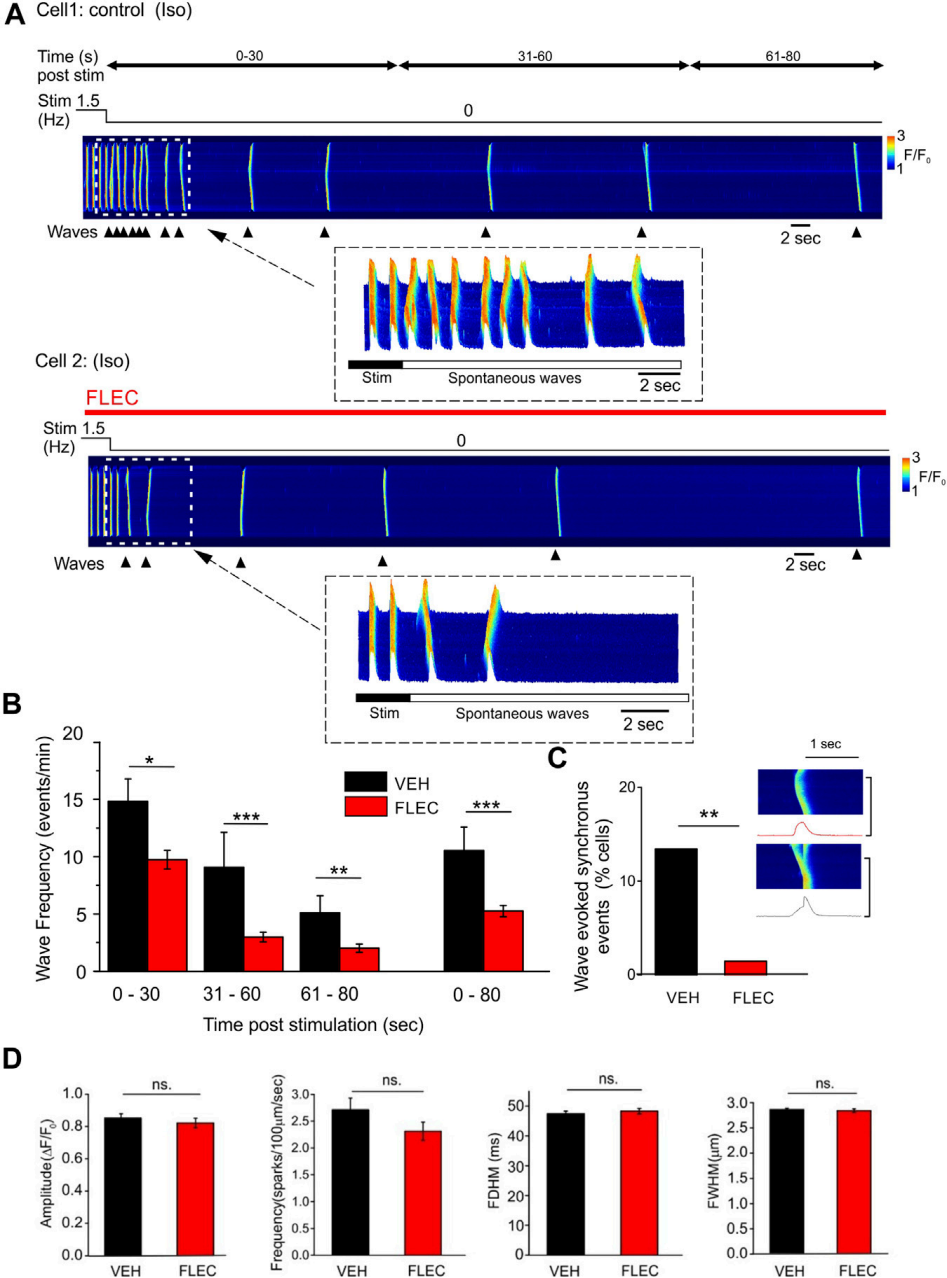
Flecainide decreases Ca^2+^ wave frequency in intact ARVM without affecting spark properties **(A)**, Representative line scan images showing spontaneous Ca^2+^ waves post-pacing (1.5 Hz) in the presence of isoproterenol (ISO) and either vehicle (VEH, *upper*) or flecainide (FLEC, *lower*). **(B)**, Cumulative data showing the mean wave frequency at various time intervals post-pacing in the presence of either vehicle or flecainide. **(C)**, Cumulative data showing the percentage of cells exhibiting in which a Ca^2+^ wave triggered a rapid uniform Ca^2+^ transient. Insert shows line scan images in the same cell of a Ca^2+^ wave (*upper*) and a wave triggering a uniform Ca^2+^ transient. **(D)**, Cumulative data showing that flecainide had no significant effect on the amplitude (ΔF/F_0_), frequency, duration (FDHM) or width (FWHM) of spontaneous Ca^2+^ sparks. * = *p* < 0.05; ** = *p* < 0.01; *** = *p* < 0.001. Control/vehicle n = 68 (10), flecainide n = 68 (10).

A line scan image is shown from a control cell in which uniform Ca^2+^ release events due to field stimulation are followed, on cessation of stimulation, by propagated Ca^2+^ waves (Figure 4A *, upper*). In the presence of flecainide, wave frequency was typically lower during the 80 s period following cessation of stimulation (Figure 4A, *lower*). The cumulative data show that wave frequency in the presence of flecainide was significantly lower than time matched control cells at all 3 selected time intervals (0–30, 31–60 and 61–80 s) after cessation of stimulation (Figure 4B). This effect exhibited a dependence on the frequency of stimulation; flecainide reduced wave frequency following stimulation at 1.5 and 2.5 Hz but not at 0.5 Hz (Supplementary Figure S1).

In some control ARVM, Ca^2+^ waves triggered rapid uniform Ca^2+^ release, most likely due to a DAD initiating an action potential (Figure 4C *, inset*). However, the cumulative data show that flecainide markedly decreased the number of cells in which waves triggered uniform Ca^2+^ transients, which is consistent with its action as an inhibitor of NaV1.5.

To investigate whether the decrease in wave frequency might be explained by a direct action of flecainide on RyR2, the properties of Ca^2+^ sparks were analyzed during the 5-s period immediately before each spontaneous Ca^2+^ wave. However, none of the characteristic flecainide induced changes in spark frequency, amplitude or width that occur in permeabilized cells (Figures 1, 2) were apparent when comparing control and drug treated intact ARVM (Figure 4D *).*

## 4 Discussion

The discovery that flecainide is effective in treating CPVT was a major advance, particularly for patients in which conventional therapy inadequately suppresses arrhythmias. However, it has been known for several decades that flecainide increases the incidence of sudden cardiac death in patients with structural heart disease (Ruskin, 1989). Consequently, a proportion of CPVT patients that are stable on flecainide therapy will become unsuitable for treatment in later life as structural heart disease presents as a comorbidity.

The detrimental effects of flecainide in structural heart disease are known to reflect its inhibitory action on NaV1.5 and consequent slowing of conduction, which leads to re-entrant arrhythmias (Coromilas et al., 1995). If flecainide’s beneficial action in CPVT results solely from NaV1.5 inhibition, opportunities for reducing comorbidity related contraindications are limited. Conversely, if flecainide’s primary action in CPVT involves a direct effect on RyR2, it may be possible to identify a flecainide-like drug with similar or greater effect on RyR2, but much less influence on Nav1.5. Hence, establishing with certainty the mechanism of flecainide’s action in CPVT is of considerable importance. The present work addresses discrepancies between findings in WT ARVM, CPVT myocytes and RyR2 channels in bilayers, which have contributed to the ongoing controversy regarding flecainide’s mechanism of action.

### 4.1 Flecainide modulates Ca^2+^ sparks and waves in permeabilized ARVM

Previous studies investigating the effects of flecainide in permeabilized rat myocytes have reported either marked effects similar to those in cells from CPVT mice (Hilliard et al., 2010) or no effect on Ca^2+^ sparks or waves (Bannister et al., 2015; Bannister et al., 2016). In the present study, where care was taken to limit laser exposure during prolonged experiments and avoid Ca^2+^ contamination, flecainide induced sustained changes in both Ca^2+^ sparks and waves in permeabilized cells (Figures 1, 2), without affecting the SR Ca^2+^ content (Supplementary Figure S2).

These effects of flecainide are unlike those of other commonly studied RyR2 inhibitors which typically increase (e.g., tetracaine) or decrease (e.g., caffeine) the SR Ca^2+^ content and have only transient effects on Ca^2+^ sparks due to a well characterized mechanism known as autoregulation (Eisner et al., 1998). The ability of flecainide to inhibit SR Ca^2+^ waves can be explained by the fact that sparks of smaller amplitude and spatial spread would be expected to reduce propagation between RyR2 clusters which are predominantly located at the junctional SR (Hilliard et al., 2010).

As the characteristic effects of flecainide on Ca^2+^ sparks described previously in CPVT myocytes can be replicated in permeabilized ARVM (Figures 1, 2), the same underlying mechanism is likely involved. Importantly, however, it was necessary to apply a much higher concentration to achieve these effects in ARVM (25 µM) compared with cells from CPVT mice (6 μM, compare with Supplementary data on (Galimberti and Knollmann, 2011).

### 4.2 No effect of flecainide on RyR2 mediated Ca^2+^ sparks in intact ARVM

Flecainide decreased the frequency of Ca^2+^ waves following cessation of pacing in the presence of isoproterenol (Figure 3A). However, there was no significant effect of flecainide on Ca^2+^ sparks in intact ARVM, even at a high extracellular drug concentration (15 µM), which exceeded the maximum level found in the plasma by at least ∼3 fold (Mano et al., 2015). Indeed, there was a trend towards a decrease in spark frequency (Figure 3D); the opposite of that observed in permeabilized cells (Figure 2). Therefore, even after prolonged exposure to high levels of extracellular flecainide, there is no evidence of a direct action on RyR2 mediated Ca^2+^ sparks in intact ARVM. In agreement with other recent studies (Sikkel et al., 2013a; Bannister et al., 2015), it seems likely that effects mediated *via* inhibition of Nav1.5 and reduced Na^+^ influx are responsible for the flecainide-induced decrease in wave frequency in intact ARVM. This conclusion is supported by the frequency dependence of wave inhibition by flecainide (Supplementary Figure S1); previous studies have demonstrated that flecainide inhibition of Nav1.5 is use/frequency dependent (Mestre et al., 2000).

Previous studies have shown that cationic drugs such as flecainide can accumulate in the heart and other organs (Conard and Ober, 1984; Latini et al., 1987). The cellular accumulation of cationic drugs occurs in part due to simple Nernstian effects, e.g., resulting from the negative charge associated with the inner surface of the sarcolemmal and mitochondrial membranes (Heller et al., 2012). The mitochondrial membrane potential is ∼3X that of the sarcolemma, which might lead to a higher concentration of cationic drugs in the mitochondria than the cytosol, as has been reported to occur with doxorubicin (Ichikawa et al., 2014). Therefore, even if there is a tendency for flecainide to accumulate within cardiac tissue, a non-uniform intracellular distribution may limit the concentration in the vicinity of RyR2. Further work is needed to investigate the intracellular distribution of flecainide in cardiac cells.

### 4.3 Countercurrent inhibition potentiates the effect of flecainide

One of the main arguments against a direct effect of flecainide on RyR2 is the apparent absence of a blocking action in isolated channels when the ionic flux is luminal to cytosolic in bilayer experiments (Bannister et al., 2015). Given the markedly different experimental conditions used in lipid bilayer experiments, it is possible that RyR2 effects present in cells cannot be observed. However, an open state blocking effect of flecainide on isolated RyR2 can be demonstrated when the inside of the SR is negatively polarized (Bannister et al., 2015). Importantly, open state block of RyR2 is entirely consistent with findings in permeabilized and intact cells, where flecainide increases spark frequency but decreases spark mass, such that the total leak and the SR content remains the same, i.e., these changes can be explained if flecainide does not impeded initial RyR2 activation, but then acts on the channels in their open state to abbreviate each spark mediated Ca^2+^ flux. As the factors that dictate the open probability of RyR2 remain constant (e.g., SR content, endogenous cytosolic regulators), this results in more but smaller Ca^2+^ sparks and no overall effect on diastolic SR Ca^2+^ leak.

How then can findings in cells and lipid bilayers be reconciled? Transient SR polarization does occur during Ca^2+^ release but is ultimately neutralized by charge compensating counter-currents (Figure 3A). In the present study, partial inhibition of the counter-current by substitution with impermeant ions produced the anticipated slowing of both Ca^2+^ uptake and release phases (Figure 3B), without affecting wave amplitude. Importantly, during countercurrent inhibition, when SR polarization is more pronounced, the effect of flecainide was increased (Figure 3E).

It has been argued that polarization of the SR is likely to be small because the countercurrent compensation is rapid, particularly that carried by RyR2 (Guo et al., 2013). However, flecainide itself inhibits the RyR2 mediated countercurrent (Bannister et al., 2015), meaning that the drug may itself potentiate SR polarization, and in turn facilitate RyR2 open state block. Such an effect would represent a novel and potentially exploitable drug mechanism. To investigate this more directly, new methods are required to allow transient changes in SR membrane potential to be measured directly.

## 5 Conclusion

This study shows that flecainide has clear effects on RyR2 in permeabilized WT ARVM, which are sustained and qualitatively similar to those reported in cells from CPVT mice. However, in permeabilized ARVM, am intracellular concentration of at least 25 µM was required to detect significant effects on RyR2. Comparable effects on Ca^2+^ sparks were not be detected in intact ARVM, even after prolonged incubation with 15 µM extracellular flecainide; the highest level that could be applied without abolishing field stimulated responses. The absence of effects on Ca^2+^ sparks can be explained if the level of intracellular flecainide is insufficient to affect RyR2. Hence, previous observations showing a lack of effect on RyR2 in ARVM cannot usefully inform about the action of the drug in cells from CPVT mice, where the sensitivity of RyR2 to flecainide is markedly higher. The potentiation of flecainide’s action on RyR2 by counter-current inhibition may have relevance to lipid bilayer studies where its blocking action on the channel required the ‘luminal’ side of the lipid bilayer to be negatively polarized.

## Data Availability

The original contributions presented in the study are included in the article/Supplementary Material, further inquiries can be directed to the corresponding authors.
