# Supplementary material for: Flecainide induces a sustained countercurrent dependent effect on RyR2 in permeabilized WT ventricular myocytes but not in intact cells
Source: Front Pharmacol. 2023 Apr 12;14:1155601. doi: 10.3389/fphar.2023.1155601 (PMC10130871; doi:10.3389/fphar.2023.1155601)
Supplement: Supplementary file 1 [file DataSheet1.PDF]

Supplementary Material

1 Supplementary Data

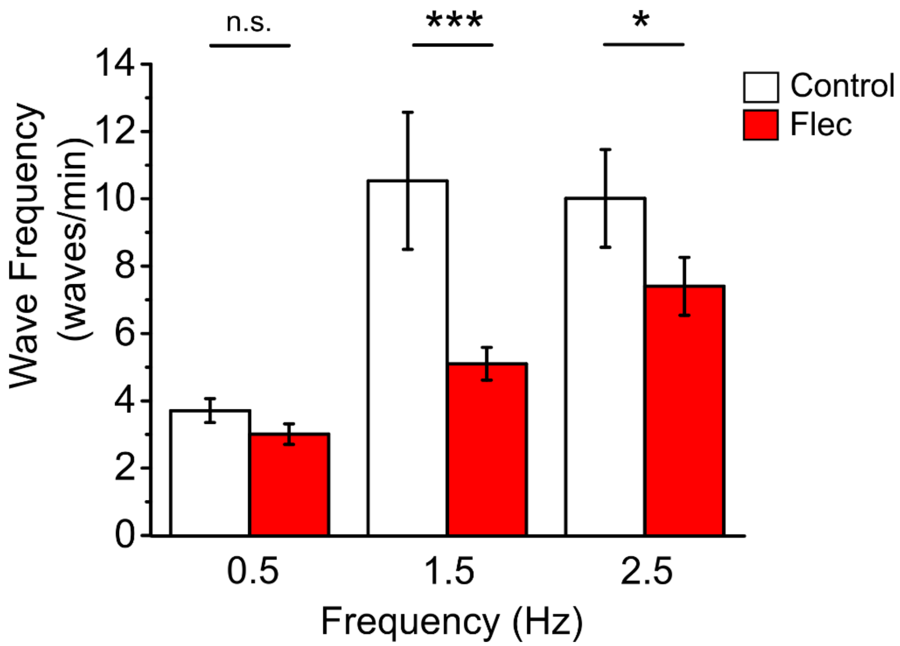

Figure S1 Frequency dependence of flecainide induced inhibition of waves in intact ARVM

Cumulative data showing the mean wave frequency at various time intervals after sudden cessation of stimulation at 0.5 1.5 or 2.5 Hz in the presence of either vehicle or flecainide (protocol as Fig. 3). * = $p < 0.05$, ***, $p<0.001$. 0.5 Hz n = 71 (6); 1.5 Hz n = 67 (10); 2.5 Hz n = 66 (9). There was no significant effect of flecainide on the frequency of waves following cessation of stimulation at 0.5 Hz (n.s.).

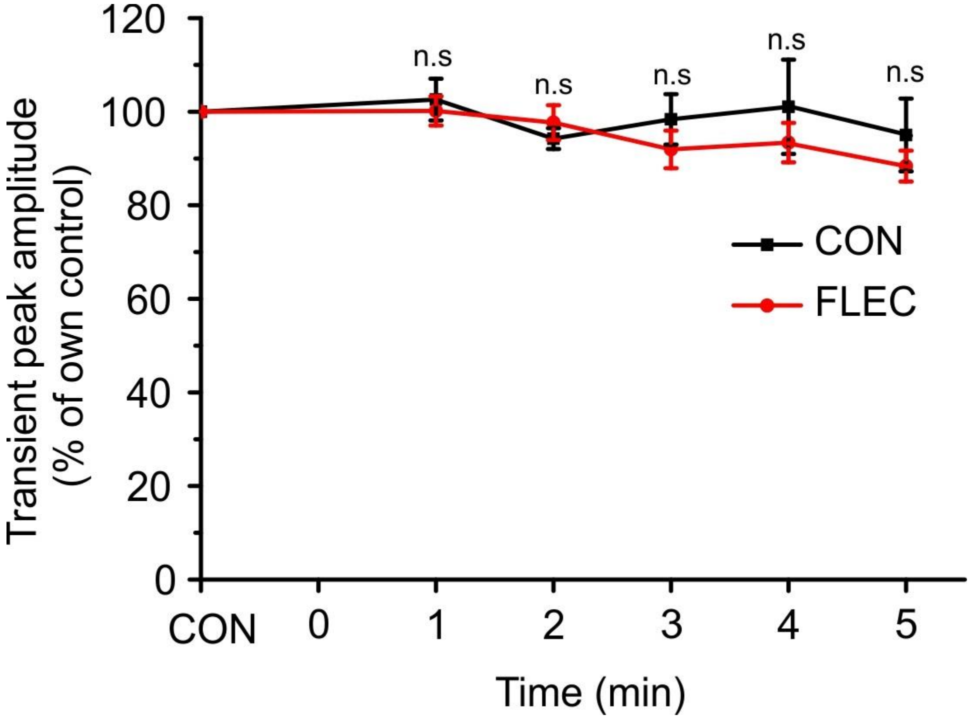

Fig. S2 Lack of effect of flecainide on SR content in permeabilized myocytes

The amplitude of the Ca^{2+} transient resulting from brief application of caffeine (20 mM) was used as an index of the SR content in the presence of flecainide (FLEC: 25 µM) or vehicle only (CON). Flecainide was added at time zero. During a continuous 5-minutes exposure, flecainide had no significant effect on the SR Ca^{2+} content at any time point. n=10(3) for both CON and FLEC. n.s. indicates $p>0.05$.
